# A gene expression atlas of adult *Schistosoma mansoni* and their gonads

**DOI:** 10.1038/sdata.2017.118

**Published:** 2017-08-22

**Authors:** Zhigang Lu, Florian Sessler, Nancy Holroyd, Steffen Hahnel, Thomas Quack, Matthew Berriman, Christoph G. Grevelding

**Affiliations:** 1 BFS, Institute of Parasitology, Justus Liebig University, 35392 Giessen, Germany; 2 Wellcome Trust Sanger Institute, Wellcome Genome Campus, Hinxton, Cambridgeshire CB10 1SA, UK

**Keywords:** Parasitic infection, Transcriptomics

## Abstract

RNA-Seq has proven excellence in providing information about the regulation and transcript levels of genes. We used this method for profiling genes in the flatworm *Schistosoma mansoni*. This parasite causes schistosomiasis, an infectious disease of global importance for human and animals. The pathology of schistosomiasis is associated with the eggs, which are synthesized as a final consequence of male and female adults pairing. The male induces processes in the female that lead to the full development of its gonads as a prerequisite for egg production. Unpaired females remain sexually immature. Based on an organ-isolation method we obtained gonad tissue for RNA extraction from paired and unpaired schistosomes, with whole adults included as controls. From a total of 23 samples, we used high-throughput cDNA sequencing (RNA-Seq) on the Illumina platform to profile gene expression between genders and tissues, with and without pairing influence. The data obtained provide a wealth of information on the reproduction biology of schistosomes and a rich resource for exploitation through basic and applied research activities.

## Background & Summary

Transcriptome profiling has substantially benefitted from sequencing approaches like as RNA-Seq. Compared to microarrays or SAGE/SuperSAGE, RNA-Seq offers a wider dynamic range and can theoretically cover all transcripts in a biological sample^1^. This was a major limitation of previous microarray approaches, where the sequences used as hybridization baits did not represent the complete genome/transcriptome, and where fluorescence intensities tend to saturate for highly expressed genes. SAGE/SuperSAGE is a RT-PCR and cloning-based technology. Although transcript quantification is possible, SAGE/SuperSAGE fails when transcripts/cDNAs lack specific restriction sites needed for cloning, or when a specific restriction site occurs too close to the polyA-tail region of an otherwise detectable mRNA/cDNA^2–7^.

RNA-Seq has previously been used to profile gene expression in parasites like schistosomes^8–13^. The latter cause schistosomiasis, a devastating infectious disease that ranks second only to malaria^14^. A vaccine is not available, and only one widely used drug (Praziquantel) exists^15^. There is a justifiable fear resistance emerging, for which experimental and field-study evidence has been obtained^16–19^. Schistosomes exhibit remarkable reproductive biology, which is not understood at the molecular level^20–23^ but may represent a vulnerability that could be exploited by novel control measures.

Schistosomes have a complex life-cycle including an intermediate snail host and a vertebrate final host in which the adult worm develops^21^. Whereas flatworms are generally hermaphrodites, schistosomes have evolved separate sexes. Sexual dimorphism is visible at the adult stage only^24^. An exceptional feature of schistosome biology, however, is the constant pairing contact which is the prerequisite for the sexual maturation of the female. Pairing induces processes in the female which lead to the differentiation of its gonads^20,21,24^. The latter comprise the ovary, producing oocytes, and the vitellarium, delivering vitelline cells that provide egg-shell proteins needed for egg production and resources for embryogenesis^20,21^. Composite eggs are finally formed within the ootype, the egg-forming organ which is connected to the ovary and vitellarium by separate ducts and additionally to the uterus which ends at the tegumental surface and ensures egg transport to the environment. The eggs possess dual capacity being important for life-cycle maintenance but also causing the pathologic consequences of schistosomiasis. Trapped in host tissues such as gut, spleen, and liver, eggs induce inflammatory processes which finally lead to liver cirrhosis^25^.

Few studies have been initiated to unravel the reproductive development of schistosomes at the molecular level^23,26–36^. Although the first molecular insights into the exceptional male-female interaction have been obtained, our knowledge is still fragmentary. To fill existing gaps we approached pairing-dependent processes and female sexual maturation by a comparative RNA-Seq analysis. To this end we isolated gonad tissue of *S. mansoni* by an organ-isolation method^37^. Adult worms were raised by rodent infections either with both genders, leading to paired worms (b; bisex), or with a single gender (s; single sex), leading to sexually naïve males (M) or virgin females (F)^38^, and RNA was extracted from complete testes (T) and ovaries (O) as well as whole worms. In this way 23 libraries of paired (bF) and unpaired (sF) females and the corresponding ovaries (bO, sO) as well as of paired (bM) and unpaired (sM) males and the appropriate testes (bT, sT) were obtained for Illumina sequencing. After mapping to the reference genome, reads were counted and used to calculate differential gene expression caused by adult pairing, gender difference, and tissue orgin.

Our data sets represent the first gonad-specific transcriptome profiles of a parasitic flatworm including genes regulated by pairing in ovaries and testes of *S. mansoni*, one of the three major species affecting humans^14,15^. In the related work^39^ published in *Scientific Reports* we performed a first in-depth bioinformatics analysis to explore and mine the data, providing an integrated overview on pairing-influenced processes that cover the majority of genes in ovaries but also genes in testes. The dataset reported here will be supportive for future studies investigating the reproductive biology of schistosomes and further parasitic flatworms, for which to our knowledge no comparable studies exist.

## Methods

Detailed methods on schistosome maintenance, gonad isolation, RNA extraction, RNA-Seq analyses and data processing can be found in our related work^39^.

### Experimental design

**Figure 1:**
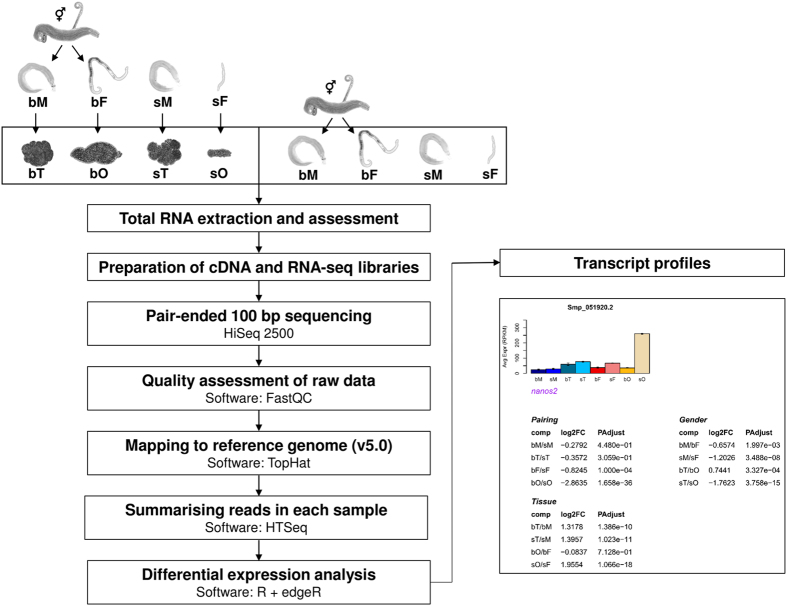
Schematic overview of the experimental design. All biological replicates (*n*=2 for sO and *n*=3 for all other samples) were obtained from the species *Schistosoma mansoni*, which was maintained in hamsters as final hosts. The separation of couples and the isolation of ovaries and testes were always performed at the same day to avoid any *in vitro*-culture effects. Total RNA was extracted by Trizol and its quality assessed by electropherogram analyses using an Agilent 2100 Bioanalyzer and Pico chips (Agilent Technologies). For each sample 100 ng of total RNA was used for synthesizing cDNA and generating libraries. Pair-end 100 bp sequencing was performed on Illumina HiSeq 2500 running two technical replicates for each sample. Raw reads were assessed using FastQC (http://www.bioinformatics.babraham.ac.uk/projects/fastqc/) and mapped to *Schistosoma mansoni* reference genome (v5.0) using TopHat. Reads for each transcript were counted using HTSeq and imported to edgeR for normalization across all samples and differential expression analysis. Mean RPKM and s.e.m. values based on normalized reads were calculated and used for barplots. A transcript profile was generated for each gene, including the barplot, as well as the log_2_ fold-change (log_2_FC) and adjusted *P* (PAdjust) values based on various comparisons (see Smp_051920.2 as representative example, a nanos-ortholog that is abundantly transcribed in ovaries of unpaired females=sO).

The workflow for the comparative RNA-Seq approach included the following steps (Fig. 1). After isolating adult worms from final hosts by perfusion^38^, paired worms were separated and their gonads isolated the same day^37,39^. All biological material was kept in Trizol in liquid nitrogen until RNA extraction. For each sample 100 ng of total RNA was used for generating RNA-Seq libraries, of which sequencing was performed with 100 bp paired-ends. After quality assessment, raw reads were mapped to *S. mansoni* reference genome using TopHat^40^ (version 2.0.8b). Differential gene expression was analyzed using the R package edgeR^41^ (v3.6.7), for which raw reads were counted by HTSeq^42^ (v0.5.4). Mean RPKM values based on normalized reads were used for illustrative purposes (barplots and heatmaps), and Standard Error of Mean (s.e.m.) values were calculated and added where applicable.

### Quantitative RT-PCR

For confirming the RNA-Seq results, real-time quantitative RT-PCRs (qPCRs) were performed. Total RNA of approved quality of each sample was used for cDNA synthesis using the QuantiTect Reverse Transcription Kit (Qiagen) including a genomic DNA wipe-out step. The subsequent qPCR was done with SYBR Green for detection (PerfeCTa SYBR Green Super Mix, Quanta) and a Rotor-Gene Q cycler (QIAGEN). Each gonad sample was analyzed with two biological replicates and two technical replicates. Ct (threshold cycle) values were obtained and compared with corresponding RPKM values from the RNA-Seq results. The online tools Primer3 Plus (http://www.bioinformatics.nl/cgi-bin/primer3plus/primer3plus.cgi/), OligoCalc (http://biotools.nubic.northwestern.edu/OligoCalc.html), and OligoAnalyzer (https://eu.idtdna.com/calc/analyzer) were applied to design and analyze primers. All primer pairs were conceived to have an annealing temperature of 60 °C. Their sequences are shown in Table 1.

### Code availability

Codes have been deposited as part of the archive in figshare (Data Citations 1, 2).

## Data Records

All sequence data can be obtained from the European Nucleotide Archive (ENA) (Data Citation 3) and from Array Express (Data Citation 4). Transcript profiles of each gene detected in the study were deposited as individual pdf files for downloading in figshare (Data Citations 1, 2). Furthermore, a web interface was created that offers immediate access to individual transcript plot data by Smp numbers of genes of interest (http://schisto.xyz/geneexp).

## Technical Validation

### Qualitative and quantitative control of extracted RNA

**Figure 2:**
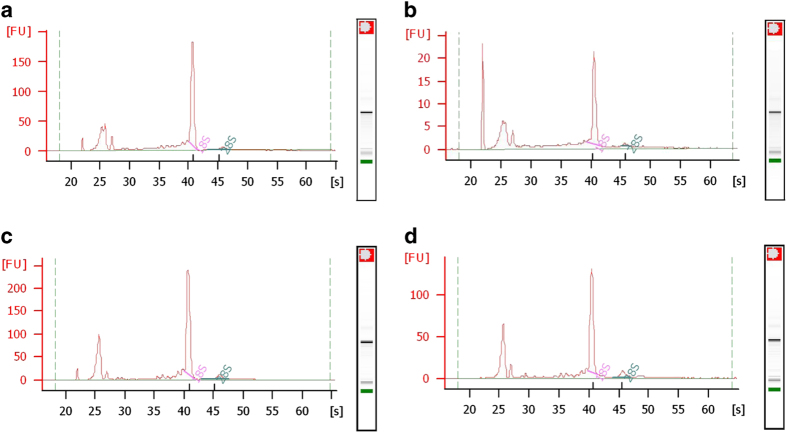
Representative results of sample RNA assessed by electropherogram analysis using an Agilent 2100 Bioanalyzer. (**a**) RNA obtained from testes of sM; (**b**) RNA obtained from ovaries of sF; (**c**) RNA obtained from sM; (**d**) RNA obtained from sF.

Quality and quantity of total RNA from each sample was checked by electropherogram analyses using the Agilent RNA 6000 Pico Kit (Agilent Technologies). Representative results of all samples are shown in Fig. 2. Note that the 28S rRNA peak is not present due to a known gap region within the molecule^43^.

### Quality control of RNA-Seq reads

**Figure 3:**
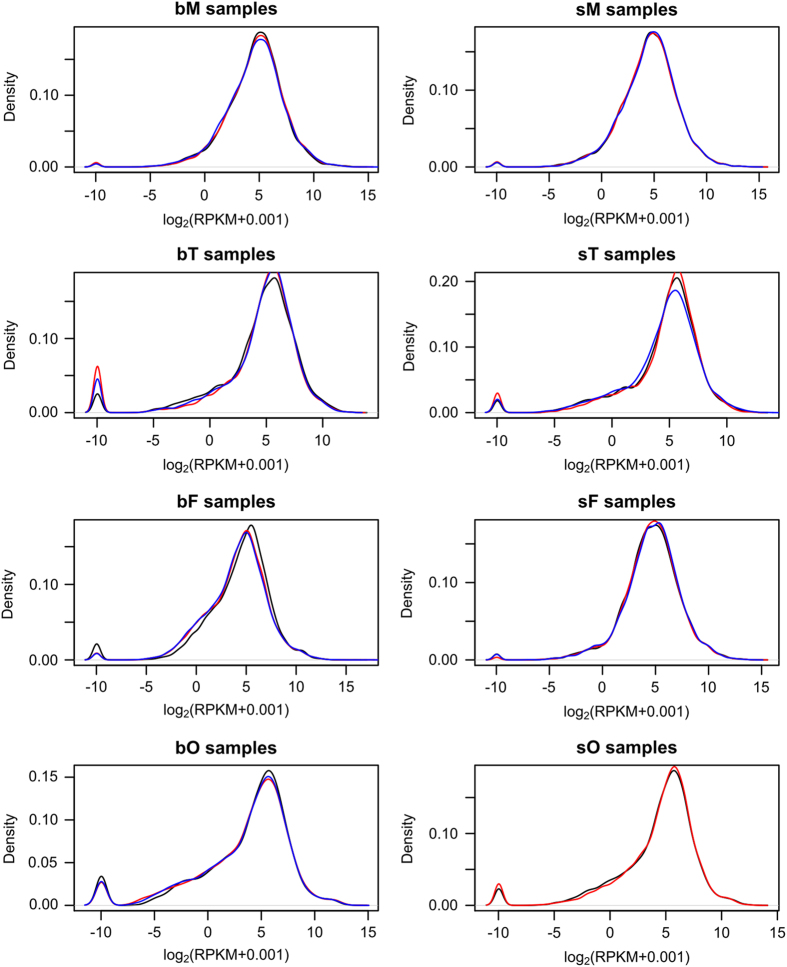
Density distribution RPKM values. Densities were calculated using log_2_(RPKM+0.001) values based on non-normalized reads. Each color (blue/red/black) represents one biological replicate.

RPKM values were calculated from non-normalized reads. Density plots were generated from transformed log_2_(RPKM+0.001) values as a method to check RPKM distributions and replicate matchings. All samples exhibited bimodal distributions, with the first peak representing the percentage of genes without any reads and the second peak showing the highest RPKM density (Fig. 3). In addition, for all eight samples, the biological replicates matched to each other indicating good correlations.

### Principal component analysis and representative transcripts

**Figure 4:**
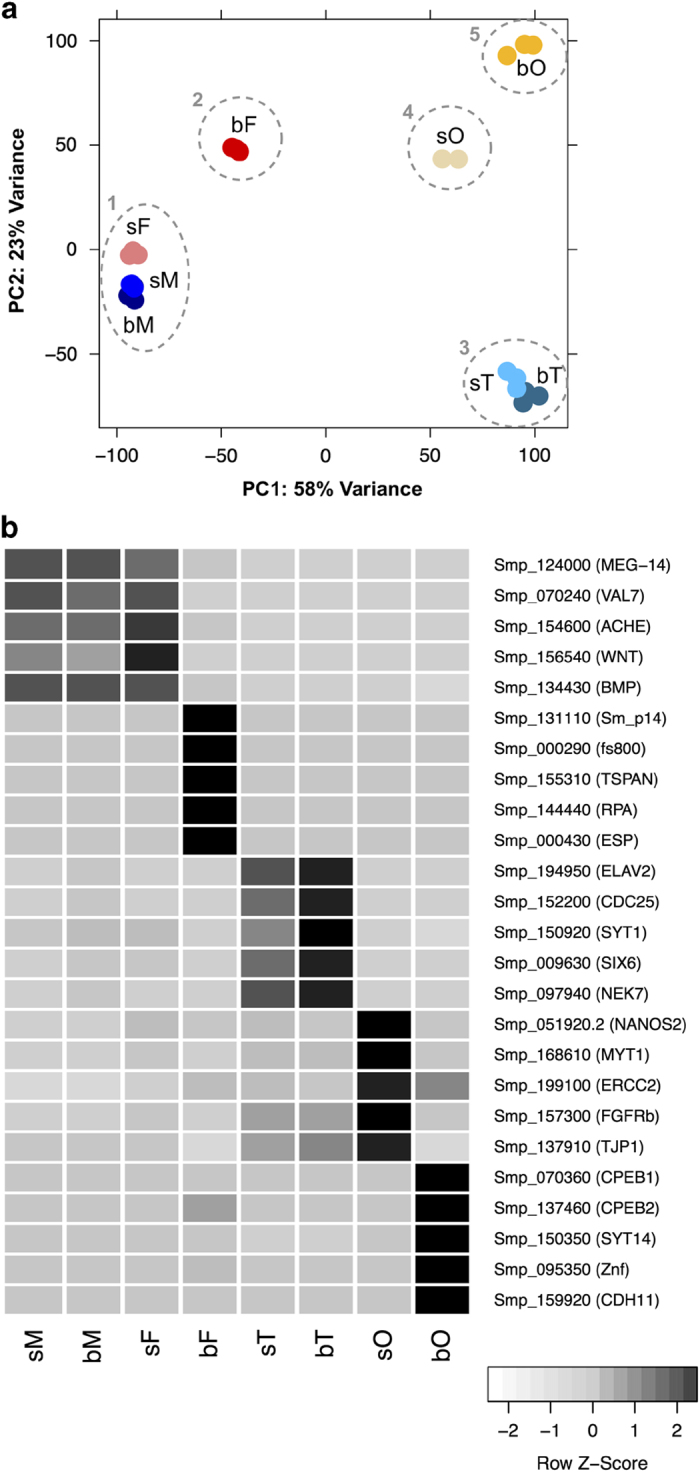
Principal Component Analysis (PCA) and representative genes for each sample cluster. (**a**) Reads for all genes were taken for PCA. Five clusters were obtained for all samples: 1) bM-sM-sF; 2) bF; 3) bT-sT; 4) sO; 5) bO. (**b**) Collection of exemplary genes found to be preferentially or specifically and pairing-dependently or -independently transcribed in either one of two adult clusters (e.g. cluster 1: Smp_124000, MEG-14; cluster 2: Smp_131110, Sm_p14), or within one of the three gonad clusters as preferentially transcribed (e.g. cluster 3: Smp_194950, ELAV2) or as specifically transcribed in one gonad type (e.g. cluster 4: Smp_051920.2, NANOS2; cluster 5: Smp_070360, CPEB1). Dark and light grey indicates high and low transcript abundance, respectively. Full names of gene symbols are as follows: MEG-14 (micro exon gene 14), VAL7 (venom allergen-like (VAL) 7 protein), ACHE (acetylcholinesterase), WNT (wnt family member), BMP (bone morphogenetic protein), Sm_p14 (eggshell protein Sm_p14), fs800 (female specific protein), TSPAN (tetraspanin CD63 receptor), RPA (Replication protein A), ESP (eggshell protein), ELAV2 (Embryonic Lethal, Abnormal Vision 2), CDC25 (M phase inducer phosphatase 3), SYT1 (synaptotagmin), SIX6 (homeobox protein SIX6), NEK7 (Serine/threonine kinase NEK7), NANOS (nanos 2), MYT1 (myelin transcription factor 1 protein), ERCC2 (DNA excision repair protein ERCC-2), FGFRb (fibroblast growth factor receptor), TJP1 (tight junction protein 1), CPEB (cytoplasmic polyadenylation element binding), SYT14 (synaptotagmin), Znf (zinc finger), CDH11 (protocadherin 11). bF, paired females; bM, paired males; bO, ovary of bF; bT, testis of bM; sF, unpaired females; sM, testis of sM; sO, ovary of sF; sT, unpaired males.

By Principle Component Analysis (PCA), all 23 samples were clustered into different groups, indicating differences and/or similarities within their transcriptomes, which except for gender or tissue were also caused by pairing. Furthermore, we identified transcription profiles that allowed conclusions about specialized gene functions, which is of high value for basic as well as applied research aspects (Fig. 4).

These examples illustrate that genes were detected with specialized pairing-dependent or -independent function in either one gender and gonad-independently, or in gonads of both genders, or gonad-specifically in one gender^39^. Although the vitellarium has not been covered as a separate reproductive female organ, genes with functions in this organ are among those differentially regulated in bF compared to sF. The vitellarium is the biggest organ in schistosome females, representing 70–80% of the body of a sexually mature female. Thus it appears likely that genes whose transcripts are more abundant in bF compared to sF, and for which few transcripts could be detected in ovaries of paired females (bO), represent genes with functions associated preferentially or specifically to the vitellarium^39^. Representative examples are p14 (Smp_131110) or fs800 (Smp_000290) (Fig. 4), both egg-shell precursor genes whose activities were demonstrated before to be specific for the vitellarium^44,45^.

### Validation of gene expression by qPCR

**Figure 5:**
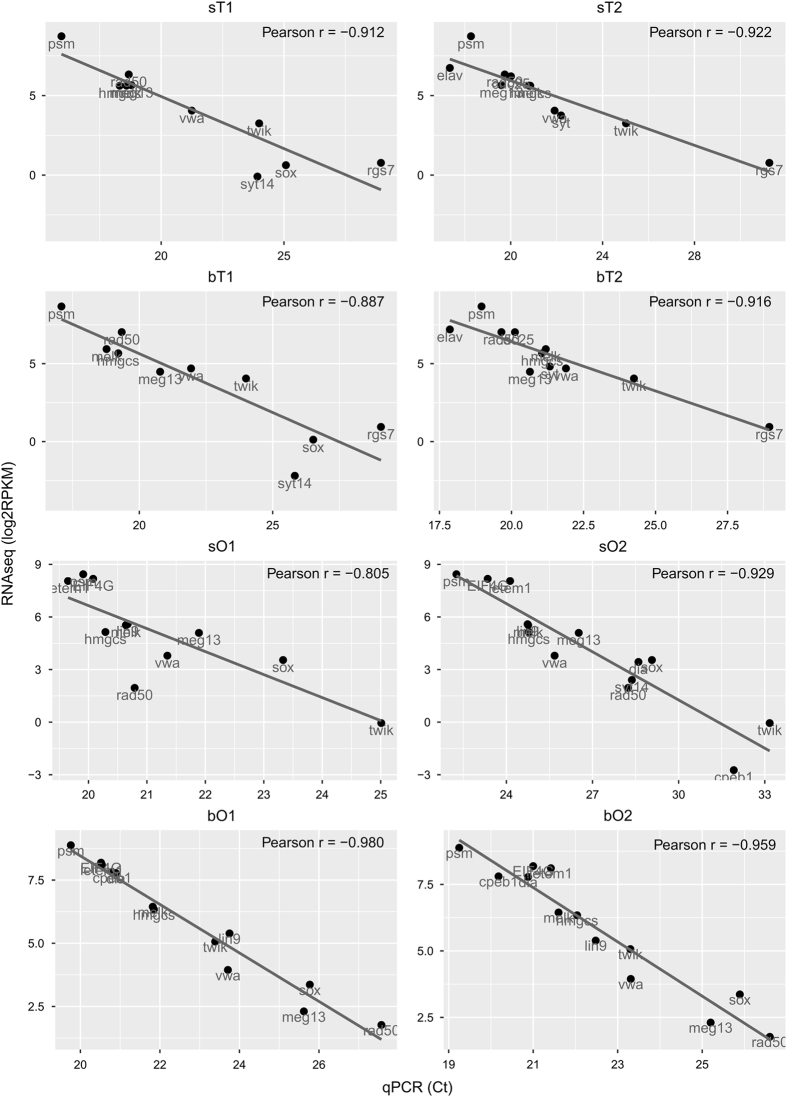
Correlations between RNA-Seq and qPCR results. Each dot indicates a single gene whose corresponding Smp-number can be found in Table 1. The selected genes include those with high (large RPKM and small Ct-values) and low transcript abundance (small RPKM and large Ct) in the gonad samples. Pearson´s correlation values are given in each case. Full names of gene symbols: psm (proteasome), rad50 (DNA double-strand break repair rad50 ATPase), melk (maternal embryonic leucine zipper kinase), hmgcs (Hydroxymethylglutaryl-CoA synthase), meg13 (micro exon gene 13), vwa (von Willebrand factor A), twik (twik family of potassium channels), sox (transcription factor SOX), syt14 (synaptotagmin XIV), cpeb1 (cytoplasmic polyadenylation element binding), dia (diaphanous), EIF4G (eukaryotic translation initiation factor 4 gamma), letem1 (LETM1 and EF hand domain containing protein 1), lin9 (protein lin9), rgs7 (regulator of G protein signaling), elav (embryonic lethal abnormal visual system), and cdc25 (M phase inducer phosphatase 3 cdc25).

Ct-values were calculated by qPCR. Pearson’s correlations between RNA-Seq expression (RPKM) and qPCR (Ct) were calculated (Fig. 5). For the analyzed genes, all gonad samples demonstrated a good correlation between RNA-Seq and qPCR expression (Pearson’s r>0.8 in all cases).

## Additional information

**How to cite this article**: Lu, Z. *et al.* A gene expression atlas of adult *Schistosoma mansoni* and their gonads. *Sci. Data* 4:170118 doi: 10.1038/sdata.2017.118 (2017).

**Publisher**’**s note**: Springer Nature remains neutral with regard to jurisdictional claims in published maps and institutional affiliations.

## Supplementary Material



## Figures and Tables

**Table 1:** List of primers used for reverse transcription and amplification of representative gene transcripts in real-time quantitative RT-PCR (qPCR) analyses.

**Gene symbol**	**Smp number**	**Forward primer (5′→3′)**	**Reverse primer (5′→3′)**	**Target length (bp)**
psm	Smp_073410	GGTCTGGTGGTTTCTCGTTC	GTACCTTCTGTTGCCCGTG	160
rad50	Smp_181450	TACCCAAAGATCTGGATGCAGA	TGAGTGGTCTAACGCATACGG	152
melk	Smp_166150	TCTCCAAGGGCTGTACCTGT	ATCAGACCCGAGCTTCCTCT	133
hmgcs	Smp_198690	GATCCTGGACTCATGTTCGC	GTACATAGCTGCTGCCATTCC	159
meg13	Smp_127990	CAAATGGATATAACTTATAGTTGGTG	TCGTTTGTGCTTGTGGAAGTAC	155
vwa	Smp_127480	TCATTATCGCTTCCATCTACCC	CAGCTGGATTATTGGTGACAGT	118
twik	Smp_147550	GGATTTGGTGATATTGTACCAGG	TTTGTGACACCTATACGACGTC	179
sox	Smp_076600	CCTTATACCACAGTTGTTGGTTC	TGTACGTTAGGTGCATCGGG	137
syt14	Smp_150350	CTGGTGGACCTTCAGCTTATC	CGACGATACGACAAATAAGCGT	168
cpeb1	Smp_070360	AGGTGGTGTTCCAATGCGTG	CTGCATAACAATAGCGTCCATC	152
dia	Smp_146810	CCGAAGACTTGAAATACGTGAG	TGAAGATGGTGATGGCTCGC	157
EIF4G	Smp_008900	GCAGGATTGAGTGACAGACG	AATCGTAAGCTTGGGACGAGA	158
letem1	Smp_065110	GAAGGTGATCAAGCTCCATTGT	TTGTACTGCATGGATAGGTGGT	162
lin9	Smp_133660	CCCAAGTTCTGTGAATGGCTG	ACGTGGTCTCCCTATGATCC	177
rgs7	Smp_210800	CAAGTACGTCAAGAGCAAATGAG	CGATGTGAAGGCAACAGTGG	119
elav	Smp_194950	GCCATCATCACCATCGAGTC	TGCTTCCAACTGATGTATCACTG	149
cdc25	Smp_152200	TACGAATCTCCTAGATCAAGGC	ACGGAGGAAGGGAACTGTG	137
